# Characteristics of the HIV cascade of care and unsuppressed viral load among gay, bisexual and other men who have sex with men living with HIV across Canada’s three largest cities

**DOI:** 10.1002/jia2.25699

**Published:** 2021-04-30

**Authors:** David M Moore, Zishan Cui, Shayna Skakoon‐Sparling, Jordan Sang, Justin Barath, Lu Wang, Nathan Lachowsky, Joseph Cox, Gilles Lambert, Syed W Noor, Daniel Grace, Jody Jollimore, Herak Apelian, Allan Lal, Abbie Parlette, Trevor A Hart

**Affiliations:** ^1^ British Columbia Centre for Excellence in HIV/AIDS Vancouver BC Canada; ^2^ University of British Columbia Vancouver BC Canada; ^3^ Ryerson University Toronto ON Canada; ^4^ University of Victoria Victoria BC Canada; ^5^ McGill University Montréal QC Canada; ^6^ Direction régionale de santé publique ‐Montréal CIUSSS Centre‐Sud‐de‐l'Ile‐de‐Montréal Montréal QC Canada; ^7^ Institut national de santé publique du Québec Montréal QC Canada; ^8^ School of Human Sciences Louisiana State University Shreveport Shreveport USA; ^9^ University of Toronto Toronto ON Canada; ^10^ Community Based Research Centre Vancouver BC Canada

**Keywords:** antiretroviral therapy, HIV, men who have sex with men, virological suppression

## Abstract

**Introduction:**

Treatment as prevention strategies have been variously applied across provinces in Canada. We estimated HIV care cascade indicators and correlates of unsuppressed viral load (VL) among gay, bisexual and other men who have sex with men (GBM) recruited in Vancouver, Toronto and Montreal.

**Methods:**

Sexually active GBM, aged ≥16 years, were recruited through respondent‐driven sampling (RDS) from February 2017 to August 2019. Participants completed a Computer‐Assisted Self‐Interview and tests for HIV and other sexually transmitted infections (STIs). We conducted bivariate analyses comparing RDS‐adjusted proportions across cities. We used multivariable logistic regression to examine factors associated with having a measured VL ≥ 200 copies/mL with data pooled from all three cities.

**Results:**

We recruited 1179 participants in Montreal, 517 in Toronto and 753 in Vancouver. The RDS‐adjusted HIV prevalence was 14.2% (95% CI 11.1 to 17.2) in Montreal, 22.1% (95% CI 12.4 to 31.8) in Toronto and 20.4% (95% CI 14.5 to 26.3) in Vancouver (*p* < 0.001). Of participants with confirmed HIV infection, 3.3% were previously undiagnosed in Montreal, 3.2% undiagnosed in Toronto and 0.2% in Vancouver (*p* = 0.154). In Montreal, 87.6% of GBM living with HIV were receiving antiretroviral therapy (ART) and 10.6% had an unsuppressed VL; in Toronto, 82.6% were receiving ART and 4.0% were unsuppressed; in Vancouver, 88.5% were receiving ART and 2.6 % were unsuppressed (*p* < 0.001 and 0.009 respectively). Multivariable modelling demonstrated that participants in Vancouver (adjusted odds ratio [AOR]=0.23; 95% CI 0.06 to 0.82), but not Toronto (AOR = 0.27; 95% CI 0.07 to 1.03), had lower odds of unsuppressed VL, compared to Montreal, as did older participants (AOR 0.93 per year; 95% CI 0.89 to 0.97), those at high‐risk for hazardous drinking (AOR = 0.19; 95% CI 0.05 to 0.70), those with a primary care provider (AOR = 0.11; 95% CI 0.02 to 0.57), and those ever diagnosed with other STIs (AOR = 0.12; 95% CI 0.04 to 0.32).

**Conclusions:**

GBM living in Montreal, Toronto and Vancouver are highly engaged in HIV testing and treatment and all three cities have largely achieved the 90‐90‐90 targets for GBM. Nevertheless, we identified disparities which can be used to identify GBM who may require additional interventions, in particular younger men and those who are without a regular primary care provider.

## 1 INTRODUCTION

Gay, bisexual and other men who have sex with men (GBM) in Canada are disproportionately affected by HIV and other sexually transmitted infections (STIs). In 2016, GBM accounted for 48% of new HIV diagnoses [1] and have consistently accounted for 45% to over 50% annually, despite comprising only 3% to 5% of the population aged 15 years and older [1, 2, 3, 4].

The use of biomedical HIV prevention has been recognized as having the potential to act as a preventive measure at both the individual level [5] and the population level [6, 7]. This approach, termed “treatment as prevention” (TasP), has been formally adopted in BC as public policy with additional dedicated funding since 2010 [8]. It is also part of the HIV control strategies in Ontario [9] and Quebec [10], but has not been developed to the same extent in these provinces. As healthcare is primarily a responsibility of provinces, rather than the federal government in Canada, policies and programmes to address HIV and other STI prevention, testing, care and treatment vary across provinces.

In 2014, UNAIDS formally proposed targets for HIV‐related health services, known as 90‐90‐90, whereby countries should aspire to have 90% of people living with HIV (PLWH) aware of their diagnosis, 90% of these individuals should be receiving ART and 90% of these should have achieved virological suppression [11]. The government of Canada formally adopted these targets in 2014 and estimated then that approximately 80% of PLWH had been diagnosed, of whom 76% were receiving ART and 89% of these had achieved virological suppression [12]. Sustained virological suppression is the ultimate goal of the care cascade in that it maximizes the benefits of ART for PLWH [13] and eliminates the risk of onward HIV transmission [5]. Previous research has identified sociodemographic factors such as age, ethnicity and income [14, 15], as being associated with virological suppression, as well as measures of mental health and substance use [15, 16, 17].

We designed a study to compare the GBM populations in Montreal, Toronto and Vancouver, by recruiting representative community‐based samples. We estimated HIV care cascade indicators across all three cities and examined factors associated with having an unsuppressed viral load (VL). Our primary interest was to examine if there were differences in HIV‐related outcomes for GBM populations in these cities and whether access to specific health services were associated with achieving virological suppression.

## 2 METHODS

Participants were recruited using respondent‐driven sampling (RDS) in Montreal, Toronto and Vancouver from 1 February 2017 to 31 August 2019. RDS is a formalized chain recruitment method [18] by which participants are recruited through their own social networks. To be eligible GBM needed to identify as a man (cis‐ or transgender), be aged ≥16 years old, report having sex with another man in the previous six months and be able to complete a survey in either English or French (if a participant from Montreal) and agree to study procedures, including biological sampling. Furthermore, participants were required to either have been invited into the study as a “seed” participant or be in receipt of a valid invitation (see below) obtained from another participant whom they knew personally. The initial targeted sample size was 720 participants per site, in order to provide comparable data for the Vancouver site with a previous RDS‐conducted study [16]. However, the Montreal site received additional funding from the Quebec government to increase its sample size to 1200 participants. All study sites began enrolment by purposely recruiting 30 seeds to initiate recruitment and study protocols allowed for the recruitment of additional seeds to maintain steady recruitment. Seeds were initially recruited through members of our Community Engagement Committees. As well. advertisements on mobile phone applications and websites used by GBM were also used to recruit additional seed participants in Toronto and Vancouver. Of the 30 initial seeds selected in each city, at least 10 were men living with HIV, 10 self‐identified as ethnic minorities, two self‐identified as transgender and two as bisexual. We also tried to recruit at least two seeds who were below the age of 18 years All seeds and subsequent recruits were provided with six invitations to offer to other GBM whom they knew personally. Invitations were either provided as a printed card or an electronic version which could be sent by text or email. Invitations had a unique identifier number to ensure that they could only be redeemed by one individual. Participants were provided with a $50 CAD honorarium for study participation and $15 CAD for each individual they recruited to participate in the study. The study samples in each city were monitored for equilibrium for key parameters including age, gender‐identity, sexual orientation, ethnicity, being born in Canada, income group and HIV serostatus.

Potential participants were screened for eligibility with a short questionnaire at each of the study offices (one in each city), and, if eligible, underwent informed consent procedures. Participants then completed a Computer‐Assisted Self‐Interview (CASI) and met with a study nurse to undergo a rapid HIV test and provided venous blood samples for serological tests for HIV, hepatitis C virus (HCV) and syphilis. For participants known to be living with HIV, we offered them the option of either confirming their diagnosis with a point‐of‐care or HIV laboratory test, or by requesting confirmation from their primary care physician. For these individuals or those who were diagnosed with HIV at the visit, the nurse also drew blood for CD4 cell counts and HIV RNA VL. The CASI collected information regarding sociodemographics, health status, use of health services and individual characteristics, including reported substance‐use. Mental health symptoms were measured by the Hospital Anxiety and Depression Scale (HADS) [19], using both the anxiety (α = 0.84) and depression (α = 0.76) sub‐scales. Alcohol use was measured by the Alcohol Use Disorders Identification Test‐Concise (AUDIT‐C) scale [20]. Study protocols were approved by institutional review boards of Research Institute of the McGill University Health Centre, Ryerson University, The University of Toronto, St. Michael’s Hospital, the University of British Columbia and the University of Victoria.

We conducted bivariate analyses comparing RDS‐adjusted proportions across cities using RDS‐II weights [21], using Pearson’s chi‐squared test for categorical variables and Type III Wald chi‐squared test (from a weighted logistic regression) for continuous variables. RDS‐II weights, which are inversely proportional to the size of participants’ social network, were based on the question “How many men who have sex with men aged 16 years or older, including trans men, do you know who live or work in the [Metro Vancouver/Greater Toronto/Metro Montreal] area?” An upper limit of network‐size was fixed at 150 based on empirical research by Dunbar *et al* [22].

Among individuals self‐reporting as HIV negative or unknown, we compared reports of their most recent HIV test and the number of tests in the previous year. For confirmed PLWH, we compared the proportions previously undiagnosed, the proportions currently receiving HIV treatment and the proportions with a plasma VL measured <200 copies/mL [23] across the three cities. We also compared sociodemographic, health service access, substance‐use behaviour and mental health symptoms among PLWH across cities. We then conducted a pooled analysis of all PLWH across all three cities using multivariable logistic regression to examine factors associated with having an unsuppressed VL (≥200 copies/mL) [23]. Variables were weighted using the RDS II‐derived weights from each city and city was used as a potential explanatory variable in the model. Variables of interest in the univariable models with a value of *p* < 0.2 were included for consideration in the multivariable model. For several variables, including age, AUDIT‐C scores and HADS scores, we considered both continuous and categorical variables for inclusion in the final model. Only observations with complete data were included in the multivariable model. There is no consensus as to whether RDS weights should be used when conducting regression analyses in RDS studies [24]. However, an analysis conducted by members of our study team found that weighted logistic regression methods consistently outperform unweighted methods in terms of bias and precision when explanatory variables are correlated with the network size reported by participants [25]. As such, we have chosen to apply RDS weights to our regression analysis. The final model was selected based on potential candidate variables from the literature and using a backward selection technique, whereby the least significant (i.e., highest Type III *p*‐value) variable was dropped until the final model reached the optimal (minimum) Akaike Information Criterion. All analyses were performed using SAS® Version 9.4 (SAS Corporation, Cary, NC, USA).

## 3 RESULTS

We recruited a total of 1179 participants in Montreal, 517 in Toronto and 753 in Vancouver, from 27, 96 and 117 seed participants respectively. A total of 6822 invitations were issued to participants in Montreal, 3078 in Toronto and 4424 in Vancouver. The mean, median and ranges of recruitment waves were 6.67, 6 and 0 to 17 in Montreal, 2.67, 2 and 0 to 12 in Toronto and 2.67, 2 and 0 to 10 in Vancouver. Further details on RDS‐specific recruitment outcomes are published elsewhere [26]. The study samples reached equilibrium for all of the key variables for which we monitored. The RDS‐adjusted HIV prevalence based on serological testing or documentation was 14.2% (95% CI 11.1 to 17.2) in Montreal; 22.1% (95% CI 12.4 to 31.8) in Toronto, and 20.4% (95% CI 14.5 to 26.3) in Vancouver (*p* < 0.001) (Table 1). Of participants who were found to be HIV negative at enrolment, 70.4% (95% CI 65.0 to 75.7) in Montreal reported having tested for HIV in the previous year, 67.5% (95% CI 57.5 to 77.6) in Toronto and 69.4% (95% CI 61.9 to 76.9) in Vancouver (*p* = 0.010). Among participants with confirmed HIV infection, 3.3% were previously undiagnosed in Montreal, with 3.2% undiagnosed in Toronto and 0.2% undiagnosed in Vancouver (*p* = 0.154). In Montreal, 87.6% of PLWH were receiving ART and 10.6% had an unsuppressed VL; in Toronto, 82.6% were receiving ART and 4.0% were unsuppressed; in Vancouver, 88.5% were receiving ART and 2.6% were unsuppressed (*p* < 0.001 and = 0.009, respectively). Notably, nine GBM living with HIV in Montreal, three in Toronto and six in Vancouver were found to have measured VLs < 200 copies/mL despite reporting that they were not receiving ART.

**Table 1:** HIV care cascade characteristics of GBM recruited in Montreal, Toronto and Vancouver in the Engage study

	Montreal (N = 1179)		Toronto (N = 517)		Vancouver (N = 753)		*p*‐value
	N (%)	RDS adjusted% (95% CI)	N (%)	RDS adjusted% (95% CI)	N (%)	RDS adjusted% (95% CI)	
HIV serostatus based on testing or documentation							
Negative/unknown	964 (81.8)	85.8 (82.8 to 88.9)	417 (80.7)	77.9 (68.2 to 87.6)	621 (82.5)	79.6 (73.7 to 85.5)	<0.001
Positive	215 (18.2)	14.2 (11.1 to 17.2)	100 (19.3)	22.1 (12.4 to 31.8)	132 (17.5)	20.4 (14.5 to 26.3)	
*Among HIV serostatus negative or unknown*							
Last tested for HIV							
Within 1 year	688 (71.4)	70.4 (65.0 to 75.7)	330 (79.3)	67.5 (57.5 to 77.6)	501 (80.7)	69.4 (61.9 to 76.9)	0.010
>1 year ago	218 (22.6)	20.5 (16.2 to 24.7)	69 (16.6)	25.2 (15.0 to 35.4)	86 (13.9)	18.3 (12.3 to 24.3)	
Never tested or unsure	58 (6.0)	9.2 (5.2 to 13.2)	17 (4.1)	7.2 (2.9 to 11.6)	34 (5.5)	12.3 (6.4 to 18.2)	
If tested within the past two years, number of times teste							
Median (IQR)	3 (2 to 5)	3 (2 to 4)	4 (2 to 6)	3 (2 to to 5)	4 (3 to 7)	4 (2 to 6)	<0.001
*Among HIV‐positive participants*							
HIV diagnosis status							
Known positive	211 (98.1)	96.7 (92.4 to 100)	98 (98.0)	96.8 (91.0 to 100)	131 (99.2)	99.8 (99.3 to 100)	0.154
Previously undiagnosed	4 (1.9)	3.3 (0.0 to 7.6)	2 (2.0)	3.2 (0.0 to 9.0)	1 (0.8)	0.2 (0.0 to 0.7)	
Use of medication for HIV							
Currently receiving HIV medication	193 (89.8)	87.6 (80.3 to 94.8)	95 (96.0)	82.6 (57.7 to 100)	123 (93.2)	88.5 (78.4 to 98.5)	<0.001
Not currently receiving HIV medication, but has in the past	4 (1.9)	1.2 (0.0 to 2.5)	0 (0.0)	0.0 (0.0 to 0.0)	3 (2.3)	6.7 (0.0 to 14.7)	
Never taken HIV medication^a^	18 (8.4)	11.2 (4.1 to 18.4)	4 (4.0)	17.4 (0.0 to 42.3)	6 (4.6)	4.8 (0.0 to 11.3)	
Plasma HIV viral load among all HIV‐positive participants							
<200 copies/mL	183 (91.0)	89.4 (82.5 to 96.4)	88 (95.7)	96.0 (89.7 to 100)	123 (96.1)	97.4 (94.6 to 100)	0.009
≥200 copies/mL	18 (9.0)	10.6 (3.6 to 17.5)	4 (4.4)	4.0 (0.0 to 10.3)	5 (3.9)	2.6 (0.0 to 5.4)	
Plasma HIV viral load among those receiving HIV medication							
<200 copies/mL	174 (94.6)	93.5 (87.2 to 99.8)	85 (97.7)	99.3 (98.2 to 100.0)	117 (96.7)	98.4 (96.3 to 100)	0.030
≥200 copies/mL	10 (5.4)	6.5 (0.2 to 12.8)	2 (2.3)	0.7 (0.0 to 1.8)	4 (3.3)	1.6 (0.0 to 3.7)	

^a^ Includes those not previously diagnosed.

Among PLWH we found differences in the age distribution across the three cities (Table 2), with Montreal having the highest RDS‐adjusted proportion of participants ≥45 years (73.5%; 95% CI 64.0 to 83.0) and the lowest proportion aged <30 (9.2%; 95% CI 1.9 to 16.5) (*p* < 0.001). Montreal had the highest proportion of participants who self‐identified as Canadian (69.7%; 95% CI 59.2 to 80.2; *p* < 0.001) and whom were born in Canada (77.6%; 95% CI 67.8 to 87.5; *p* = 0.003) with Vancouver having the lowest proportion reporting Canadian ethnicity (41.8%; 95% CI 26.3 to 57.4; *p* < 0.001) and Toronto having the lowest proportion born in Canada (62.1%; 95% CI 36.2 to 88.0; *p* = 0.003). In terms of health services, the proportions of PLWH who reported having access to a family doctor, ranged from 90.3% in Montreal to 99.4% in Toronto (*p* < 0.001). The median number of times participants had been tested for an STI in the past 2 years was highest in Toronto (5; Interquartile range [IQR] 3 to 8) and lowest in Vancouver (median of 2; IQR 1 to 5*, p = *0.022), but differences in the proportion of participants who had ever been diagnosed with an STI (other than HIV) were not statistically significant (range of 80.3% to 88.4% across sites; *p* = 0.224).

**Table 2:** Characteristics of HIV positive participants in the Engage Study by recruitment site

	Montreal (N = 215)		Toronto (N = 100)		Vancouver (N = 132)		*p* value
	N (%)	RDS% (95% CI)	N (%)	RDS% (95% CI)	N (%)	RDS% (95% CI)	
*Sociodemographics*							
Age							
<30	13 (6.1)	9.2 (1.9 to 16.5)	24 (24.0)	28.0 (7.3 to 48.7)	10 (7.6)	12.3 (2.4 to 22.2)	<0.001
30 to 44	56 (26.1)	17.3 (10.1 to 24.5)	40 (40.0)	27.6 (3.4 to 51.8)	44 (33.3)	27.1 (12.9 to 41.2)	
45+	146 (67.9)	73.5 (64.0 to 83.0)	36 (36.0)	44.4 (17.0 to 71.9)	78 (59.1)	60.6 (44.7 to 76.5)	
Annual income							
<$30,000	149 (69.3)	70.4( 59.8 to 81.0)	51 (51.0)	46.3 (19.9 to 72.8)	86 (65.2)	83.8 (75.8 to 91.7)	<0.001
$30,000 to $59,999	52 (24.2)	24.7 (14.4 to 35.1)	31 (31.0)	50.0 (23.1 to 76.9)	28 (21.2)	11.0 (4.4 to 17.7)	
$60,000+	14 (6.5)	4.9 (0.9 to 8.9)	18 (18.0)	3.7 (0.7 to 6.6)	18 (13.6)	5.2 (1.7 to 8.7)	
Ethnicity							
Canadian	161 (74.9)	69.7 (59.2 to 80.2)	46 (46.0)	46.6 (19.4 to 73.8)	76 (57.6)	41.8 (26.3 to 57.4)	<0.001
European	19 (8.8)	9.8 (3.4 to 16.2)	21 (21.0)	9.8 (1.5 to 18.0)	18 (13.6)	13.5 (2.5 to 24.5)	
Aboriginal	2 (0.9)	0.1 (0.0 to 0.3)	0 (0.0)	0.0 (0.0 to 0.0)	11 (8.3)	13.1 (0.0 to 30.5)	
Asian	3 (1.4)	3.9 (0.0 to 10.3)	6 (6.0)	21.8 (0.0 to 47.9)	7 (5.3)	13.0 (1.8 to 24.1)	
African/Caribbean/Black	8 (3.7)	4.9 (0.0 to 10.0)	8 (8.0)	4.0 (0.0 to 8.1)	3 (2.3)	6.1 (0.0 to 15.5)	
Mixed race	4 (1.9)	0.9 (0.0 to 2.1)	3 (3.0)	3.2 (0.0 to 7.5)	3 (2.3)	3.7 (0.0 to 8.9)	
Other	18 (8.4)	10.7 (4.3 to 17.0)	16 (16.0)	14.5 (0.0 to 29.4)	14 (10.6)	8.8 (1.3 to 16.3)	
Born in Canada							
No	36 (16.7)	22.4 (12.5 to 32.2)	34 (34.0)	37.9 (12.0 to 63.8)	38 (28.8)	36.4 (20.8 to 51.9)	0.003
Yes	179 (83.3)	77.6 (67.8 to 87.5)	66 (66.0)	62.1 (36.2 to 88.0)	94 (71.2)	63.6 (48.1 to 79.2)	
Sexual identity							
Gay	186 (86.5)	83.1 (74.6 to 91.7)	86 (86.0)	75.6 (49.7 to 100.0)	117 (88.6)	72.1 (53.5 to 90.6)	<0.001
Bisexual	14 (6.5)	8.6 (2.5 to 14.8)	6 (6.0)	22.8 (0.0 to 48.8)	5 (3.8)	11.6 (0.0 to 23.4)	
Other	15 (7.0)	8.2 (1.7 to 14.8)	8 (8.0)	1.6 (0.0 to 3.3)	10 (7.6)	16.3 (0.0 to 34.0)	
Gender identity							
Cis‐gender	199 (92.6)	91.4 (85.1 to 97.7)	98 (98.0)	99.0 (97.2 to 100)	121 (91.7)	86.1 (68.6 to 100)	0.002
Trans or other	16 (7.4)	8.6 (2.3 to 14.9)	2 (2.0)	1.0 (0.0 to 2.8)	11 (8.3)	13.9 (0.0 to 31.4)	
Education level							
High school or less	67 (31.2)	31.1 (20.9 to 41.3)	15 (15.0)	25.4 (0.0 to 51.2)	31 (23.5)	38.8 (20.8 to 56.8)	0.086
Greater than high school	148 (68.8)	68.9 (58.7 to 79.1)	85 (85.0)	74.6 (48.8 to 100)	101 (76.5)	61.2 (43.2 to 79.2)	
Employed							
No	106 (49.3)	53.9 (42.7 to 65.1)	43 (43.0)	62.2 (39.1 to 85.3)	61 (46.2)	64.3 (49.7 to 78.9)	0.121
Yes	109 (50.7)	46.1 (34.9 to 57.3)	57 (57.0)	37.8 (14.7 to 60.9)	71 (53.8)	35.7 (21.1 to 50.3)	
*Health services*							
Has a primary healthcare provider							
No	18 (8.4)	9.7 (3.3 to 16.1)	2 (2.0)	0.6 (0.0 to 1.5)	7 (5.3)	1.4 (0.1 to 2.8)	<0.001
Yes	197 (91.6)	90.3 (83.9 to 96.7)	98 (98.0)	99.4 (98.5 to 100)	125 (94.7)	98.6 (97.2 to 99.9)	
Number of times tested for STIs in past two years							
Median (IQR)	4 (2 to 7)	3 (2 to 6)	4 (3 to 7)	5 (3 to 8)	4 (2 to 8)	2 (1 to 5)	0.022
Ever diagnosed with an STI							
No	26 (12.2)	17.0 (8.8 to 25.2)	7 (7.1)	19.7 (0.0 to 44.6)	9 (7.1)	11.6 (2.3 to 20.9)	0.224
Yes	188 (87.9)	83.0 (74.8 to 91.2)	92 (92.9)	80.3 (55.4 to 100)	118 (92.9)	88.4 (79.1 to 97.7)	
*Substance use*							
AUDIT‐C scale score							
Low risk (score <4)	111 (55.0)	59.5 (48.3 to 70.8)	56 (57.1)	69.5 (48.8 to 90.1)	77 (62.1)	73.0 (60.0 to 86.1)	0.032
High risk (score ≥4)	91 (45.1)	40.5 (29.2 to 51.7)	42 (42.9)	30.5 (9.9 to 51.2)	47 (37.9)	27.0 (13.9 to 40.0)	
Methamphetamine use in past six months							
No	145 (71.1)	75.8 (65.7 to 85.8)	63 (63.0)	86.3 (77.1 to 95.5)	79 (59.9)	63.1 (47.8 to 78.5)	<0.001
Yes	59 (28.9)	24.2 (14.2 to 34.3)	37 (37.0)	13.7 (4.5 to 22.9)	53 (40.2)	36.9 (21.5 to 52.2)	
Injection drug use in past six months							
No	174 (83.3)	82.6 (73.5 to 91.6)	83 (83.0)	94.2 (89.5 to 98.9	109 (82.6)	89.0 (80.4 to 97.7)	0.013
Yes	35 (16.8)	17.4 (8.4 to 26.5)	17 (17.0)	5.8 (1.1 to 10.5)	23 (17.4)	11.0 (2.3 to 19.6)	
Cocaine use in past six months							
No	159 (78.7)	81.9 (73.6 to 90.3)	74 (74.8)	92.3 (86.5 to 98.0)	102 (78.5)	81.6 (69.0 to 94.2)	0.043
Yes	43 (21.3)	18.1 (9.7 to 26.4)	25 (25.3)	7.7 (2.0 to 13.5)	28 (21.5)	18.4 (5.8 to 31.0)	
Ecstasy use in past six months							
No	158 (77.1)	82.1 (74.1 to 90.1)	76 (76.8)	92.6 (86.9 to 98.4)	96 (74.4)	79.8 (66.7 to 92.8)	0.021
Yes	47 (22.9)	17.9 (9.9 to 25.9)	23 (23.2)	7.4 (1.6 to 13.1)	33 (25.6)	20.2 (7.2 to 33.3)	
Opioid use in past six months							
No	178 (87.7)	89.7 (83.2 to 96.2)	90 (91.8)	97.7 (95.3 to 100)	114 (87.7)	83.3 (71.2 to 95.3)	0.002
Yes	25 (12.3)	10.3 (3.8 to 16.8)	8 (8.2)	2.3 (0.0 to 4.7)	16 (12.3)	16.7 (4.7 to 28.8)	

In terms of substance use, Montreal had the highest proportion of participants classified at high risk for heavy drinking or abuse/dependence (40.5%; 95% CI 29.2 to 51.7) (*p* = 0.032), whereas Vancouver had the highest proportion of participants reporting methamphetamine use in the past six months (36.9%; 95% CI 21.5 to 52.2) (*p* < 0.001). Vancouver also had the highest proportion of reported opioid use in the previous six months, compared with 2.3% (95% CI 0.0 to 4.7) in Toronto and 10.3% in Montreal (95% CI 3.8 to 16.8) (*p* = 0.002).

Among all 421 PLWH in the three cities with valid VL results, 27 (6.4% non‐RDS adjusted) had measured VLs > 200 copies/mL. Our multivariable model (Table 3), based on 398 observations with complete data found that older age was associated with lower odds of unsuppressed VL (adjusted odds ratio [AOR] 0.93 per year; 95% CI 0.89 to 0.97), as was residing in Vancouver (AOR = 0.23; 95% CI 0.06 to 0.82) relative to Montreal. Toronto also had a lower odds compared to Montreal, but this was not statistically significant (AOR = 0.27; 95% CI 0.07 to 1.03). Having a primary care provider (AOR = 0.11; 95% CI 0.02 to 0.57), and ever being diagnosed with another STI (AOR = 0.12; 95% CI 0.04 to 0.32) also had lower odds of an unsuppressed VL. Participants with high‐risk scores AUDIT‐C scores, also had lower odds of having an unsuppressed VL (AOR = 0.19; 95% CI 0.05 to 0.70). No measures of mental health symptoms or diagnoses were retained in the final multivariable model.

**Table 3:** Logistic regression analysis of factors associated with having a VL ≥ 200 copies/mL among 421 participants living with HIV in the Engage Study

	Univariable logistic			Multivariable logistic		
	Odds ratio (OR	95% CI		Adjusted OR	95% CI	
Demographics						
Age	0.94	0.91	0.97	0.93	0.89	0.97
Annual income						
<$30,000	Ref					
≥$30,000	1.04	0.45	2.39			
Ethnicity – Canadian or European						
Yes	Ref					
No	1.06	0.44	2.56			
Born in Canada						
No	Ref					
Yes	0.52	0.23	1.17			
Education level						
High school or less	Ref					
Greater than high school	2.05	0.76	5.52			
Currently employed						
No	Ref					
Yes	0.91	0.41	1.99			
City						
Montreal	Ref			Ref		
Toronto	0.36	0.12	1.10	0.27	0.07	1.03
Vancouver	0.22	0.07	0.73	0.23	0.06	0.82
Sexual behaviour						
No anal sex in P6 M	Ref					
No reported CAS in P6 M	0.49	0.16	1.54			
Only reported CAS with same serostatus partners in P6 M	0.02	0.00	3.53			
Reported CAS with opposite or unknown serostatus partners	0.77	0.28	2.12			
Health services						
Has a primary healthcare provider						
No	Ref			Ref		
Yes	0.08	0.02	0.25	0.11	0.02	0.57
Tested for STIs in past two years						
No	Ref					
Yes	1.12	0.44	2.82			
Ever diagnosed with an STI						
No	Ref			Ref		
Yes	0.16	0.07	0.35	0.12	0.04	0.32
Mental health						
HADS score anxiety sub‐scale	1.11	1.03	1.19			
HADS score depression sub‐scale	1.09	0.99	1.18			
Self‐reported mental health in past six months						
Excellent/very good	Ref					
Good	1.27	0.38	4.26			
Fair/poor	3.73	1.54	9.05			
Previously diagnosed with anxiety disorder						
No	Ref.					
Yes	2.41	1.06	5.45			
Previously diagnosed with depressive or bipolar disorder						
No	Ref					
Yes	1.74	0.73	4.16			
Substance use						
Has used marijuana in past six months						
No	Ref.					
Yes	0.61	0.27	1.38			
Has used methamphetamines in past six months						
No	Ref					
Yes	1.04	0.41	2.64			
Has used injection drugs in past six months						
No						
Yes	0.66	0.15	2.79			
Previously diagnosed with substance use disorder						
No	Ref					
Yes	1.00	0.34	2.96			
AUDIT C scale						
Low risk (score <4)	Ref			Ref		
High risk (score ≥4)	0.23	0.07	0.75	0.19	0.05	0.70

CAS, condomless anal sex; HADS, Hospital Anxiety and Depression Scale.

## 4 DISCUSSION

Our RDS‐weighted analysis has demonstrated that GBM in Montreal, Toronto and Vancouver are highly engaged in HIV testing and treatment. While we did find some statistically significant differences across the three cities, the magnitude of these differences was quite small. We found that PLWH in Vancouver had lower odds of having an unsuppressed VL, compared with Montreal, but even in Montreal, only 10% of PLWH (whether diagnosed or not) had VL measurements ≥200 copies/mL. This suggests that policies and services to engage GBM at risk for acquiring HIV and those living with HIV have been largely successful across all three cities. In terms of the UNAIDS targets for this population, 97% to 99.8% of GBM living with HIV in our study were diagnosed, 82% to 89% of those were receiving treatment, and of those receiving ART, 94% to 99% had a suppressed VL. While the ART uptake values were all below 90%, we suspect that this may be due to underreporting of treatment status, as 18 participants were found to have a suppressed VL despite reporting not being on treatment.

Between 67.5 and 70.4% of HIV‐negative or unknown serostatus participants reported having been tested for HIV in the past year, and only 7% to 12% of participants reported never being tested. Previous research has shown that, in Vancouver, less frequent HIV testing among GBM is associated with less HIV risk behaviour [27]. As such, previously undiagnosed HIV infections were very rare in the current study, ranging 0.2% of PLWH in Vancouver to 3% in Montreal and Toronto. These proportions of undiagnosed infections are far lower than the national estimate of undiagnosed HIV infections (13%) released by the Public Health Agency of Canada (PHAC) in 2018 [28]. However, these PHAC estimates do not disaggregate by HIV exposure category and no primary data collection which could be used to measure the undiagnosed fraction in GBM has been published in Canada in more than 10 years [29]. While it is reasonable to assume that GBM living in these metropolitan areas are perhaps more engaged in HIV and STI care than other GBM in these provinces, it is also important to note that HIV is highly concentrated in the urban cores of these three cities in each province, with over 70% of HIV diagnoses found in these metropolitan areas being among GBM [30, 31, 32]. Notably PHAC estimates for the proportion of all PLWH receiving ART (85%) and those with virological suppression (94%) [26] are similar to what we found in our study, for GBM living with HIV.

When examining the pooled analysis of GBM living with HIV with unsuppressed VL, we found that, similar to other North American studies, younger GBM living with HIV were less likely to have a suppressed VL [14, 15]. Of note, unlike recent studies from the United States [15, 17], but similar to another study from Canada [14], we did not find that ethnicity was significantly associated with lack of viral suppression in this analysis. However, we had limited statistical power to examine associations with any single minority group, and our heterogeneous comparison of European/Canadian to all other‐identifying GBM may mask differential experiences given the diversity of minority groups that were represented in our sample.

With respect to health services, having a primary care provider and ever having been diagnosed with an STI were both associated with reduced odds of unsuppressed VL. In all three cities, much HIV care is provided by family physicians, who also manage other acute and chronic conditions. Even in circumstances where specialists may be providing HIV‐specific care, having a primary care provider likely facilitates a continuity of healthcare that benefits of PLWH. Similarly, having been previously diagnosed with an STI likely reflects access and motivation to undertake STI testing, where questions regarding HIV treatment and adherence may also be addressed at the same time. As well, men who have been previously diagnosed with an STI may be more motivated to adhere to ART recognizing that they may be at greater risk of onward transmission if not adherent to their ART.

Other studies have found that syndemic factors related to mental health and substance use are associated with a lack of HIV virological suppression among GBM [15, 17]. In our study, we did not find independent associations between measures of mental health symptoms or diagnosed disorders and lack of VL suppression, although some measures were associated with this outcome in univariable analyses. This suggests that other factors in our model, possibly having access to a family doctor or older age may explain the lack of these associations in our final model.

We also did not find associations with recent use of methamphetamine, cocaine, ecstasy, opioids or any injection drugs and our outcome. We did, however, find an association with high‐risk AUDIT‐C scores and a reduced odds of unsuppressed VL, which was unexpected. In a systematic review of the effects of alcohol‐use disorder (AUD) on HIV treatment adherence among PLWH in general (not only GBM), five of seven prospective cohort studies found an association between AUD and reduced adherence to treatment and five other studies have found associations between AUD and increased VL [33]. It is worth noting that not all studies which have examined these outcomes have found associations between AUD and HIV‐related outcomes [34] and there are wide variations as to how alcohol use and treatment adherence are measured, but we are unaware of other studies showing that high‐risk consumption may be protective. GBM who had scores for high‐risk drinking on the full AUDIT scale in Vancouver have been shown to have higher levels of social support and are more likely to read gay newspapers or community magazines [30]. This may lead such men who are living with HIV to be more aware and more motivated to seek HIV treatment and adhere to their prescribed regimens.

This study has a number of strengths, as well as several limitations. First, we used RDS as our recruitment strategy to overcome of some of the limitations of generating representative research samples of GBM in Canadian cities, which have typically used clinic‐based samples, convenience samples, or on‐line surveys or time‐location sampling from venues or events [29]. Furthermore, the study teams in each city used harmonized study protocols with the same general recruitment strategies, study procedures and data collection tools. Nevertheless, study implementation varied by city, with Montreal able to recruit a larger sample, with fewer seed participants and more waves of recruitment than Toronto and Vancouver. As such, some of the differences we have observed between the city samples may be due to differences in the characteristics arising from differences in implementation. We have conducted analyses to identify differences in sociodemographic characteristics or motivations for study participation among participants in the study across the three cities [35], but have been unable to identify reasons as to why the RDS process was more successful in Montreal than the other two cities As well, conducting pooled regression analyses using RDS‐recruited samples, violates one of the key assumptions of RDS [21], in that the participants are drawn from a distinct networked population. However, our pooled analysis did not seek to ascertain pooled prevalence estimates; instead, we sought to examine correlates of unsuppressed viral load.

## 5 CONCLUSIONS

We found that GBM living with HIV in Canada’s three largest population centres were rarely unaware of their HIV status and are highly engaged in HIV treatment. Nevertheless, we have identified disparities which can be used to identify GBM who may require additional interventions to maximize the benefits of HIV treatment, in particular younger men and those who may not have a regular primary care provider. GBM populations continue to be a core group in Canada’s concentrated HIV epidemic and while improvements in HIV prevention, care and treatment are still possible, it appears that HIV policies and programmes in these three cities have been effective in reaching the 90‐90‐90 targets. This bodes well for future reductions in HIV diagnoses (and incidence) for GBM, a phenomenon which is currently being observed in BC, where the number of diagnoses among GBM recorded in 2017 was the lowest since the mid‐1980s [30]. However, this also suggests that further improvements to the HIV care cascade, possibly 95‐95‐95 and the expansion of publicly funded pre‐exposure prophylaxis programmes [36] for HIV, which is still limited in Canada [37] may be needed to further reduce HIV infections for GBM.

## Competing interests

None of the authors have any competing interests to declare.

## Authors’ contributions

DMM, NL, JC, GL, JJ and TAH designed the study. HA, AL and AL supervised data collection and study implementation. JB was responsible for managing the study database and developed the analytic dataset. ZC and LW conducted the analyses. SWN, SSS and JS helped to develop the analysis plan. DMM developed the first draft of the manuscript and all authors provided input into and have approved the final version.

## References

[1] Bourgeois Ac , Edmunds M , Awan A , Jonah L , Varsaneux O , Siu W . HIV in Canada—surveillance report, 2016. Canada Commun Dis Rep. 2017;43(12):248–56. 10.14745/ccdr.v43i12a01 PMC5764722 29770056

[2] Public Health Agency of Canada . A PAN‐CANADIAN FRAMEWORK FOR ACTION: Reducing the Health Impact of Sexually Transmitted and Blood‐Borne Infections in Canada by 2030. Government of Canada. 2018.

[3] Statistics Canada . Census in Brief: Same‐sex couples in Canada in 2016 2017 Aug 2018. [cited 2020 Sep 22]. Available from: https://www12.statcan.gc.ca/census‐recensement/2016/as‐sa/98‐200‐x/2016007/98‐200‐x2016007‐eng.cfm

[4] Lee H , Armstrong HL , Cox J , Lambert G , Hart TA , Kroch A , et al. Trends in HIV diagnoses by age and ethnicity among men who have sex with men (MSM) in British Columbia, Ontario, and Quebec: 2006‐2015. In 27th Annual Canadian Conference on HIV/AIDS Research. 2018. Vancouver, BC, Canada.

[5] Rodger AJ , Cambiano V , Bruun T , Vernazza P , Collins S , Degen O , et al. Risk of HIV transmission through condomless sex in serodifferent gay couples with the HIV‐positive partner taking suppressive antiretroviral therapy (PARTNER): final results of a multicentre, prospective, observational study. Lancet. 2019;393(10189): 2428–38. 31056293 10.1016/S0140-6736(19)30418-0 PMC6584382

[6] Montaner JS , Lima VD , Harrigan PR , Lourenço L , Yip B , Nosyk B , et al. Expansion of HAART coverage is associated with sustained decreases in HIV/AIDS morbidity, mortality and HIV transmission: the "HIV Treatment as Prevention" experience in a Canadian setting. PLoS One. 2014;9:e87872. 24533061 10.1371/journal.pone.0087872 PMC3922718

[7] Das M , Chu PL , Santos G‐M , Scheer S , Vittinghoff E , McFarland W , et al. Decreases in community viral load are accompanied by reductions in new HIV infections in San Francisco. PLoS One. 2010;5:e11068. 20548786 10.1371/journal.pone.0011068 PMC2883572

[8] British Columbia Ministry of Health . *From hope to health: towards and AIDS‐free generation* . 2012.

[9] Ontario Advisory Committee on HIV/AIDS . *Focusing our efforts: Changing the course of the HIV prevention, engagement and care cascade in Ontario* . 2016, Ontario Ministry of Health and Long‐Term Care.: Toronto, Ontario.

[10] La Direction des communications du ministère de la Santé et des Services sociaux La thérapie antirétrovirale pour les adultes infectés par le VIH ‐ Guide pour les professionnels de la santé du Québec. 2016.

[11] UNAIDS . *90‐90‐90 An ambitious treatment target to help end the AIDS epidemic* . 2014.

[12] Public Health Agency of Canada . *Summary: Measuring Canada's progress on the 90‐90‐90 HIV targets* . 2016.

[13] DHHS Panel on Antiretroviral Guidelines for Adults and Adolescents — A Working Group of the Office of AIDS Research Advisory Council (OARAC) Guidelines for the use of antiretroviral agents in adults and adolescents with HIV. AIDSinfo. 2019.

[14] Tanner Z , Lachowsky N , Ding E , Samji H , Hull M , Cescon A , et al. Predictors of viral suppression and rebound among HIV‐positive men who have sex with men in a large multi‐site Canadian cohort. BMC Infect Dis. 2016;16(1):590. 27769246 10.1186/s12879-016-1926-z PMC5073906

[15] Sheehan DM , Dawit R , Gbadamosi SO , Fennie KP , Li T , Gebrezgi M , et al. Sustained HIV viral suppression among men who have sex with men in the Miami‐Dade County Ryan White Program: the effect of demographic, psychosocial, provider and neighborhood factors. BMC Public Health. 2020;20(1):326. 32169065 10.1186/s12889-020-8442-1 PMC7069036

[16] Moore DM , Cui Z , Lachowsky N , Raymond HF , Roth E , Rich A , et al. HIV community viral load and factors associated with elevated viremia among a community‐based sample of men who have sex with men in Vancouver, Canada. J Acquir Immune Defic Syndr. 2016;72(1):87–95. 26825177 10.1097/QAI.0000000000000934 PMC4837069

[17] Friedman MR , Stall R , Silvestre AJ , Wei C , Shoptaw S , Herrick A , et al. Effects of syndemics on HIV viral load and medication adherence in the multicentre AIDS cohort study. AIDS. 2015;29(9):1087–96. 25870981 10.1097/QAD.0000000000000657 PMC4739626

[18] Heckathorn DD . Respondent‐driven sampling: a new approach to the study of hidden populations. Soc Probl. 1997;44(2):174–99.

[19] Zigmond AS , Snaith RP . The hospital anxiety and depression scale. Acta Psychiatr Scand. 1983;67:361–70. 6880820 10.1111/j.1600-0447.1983.tb09716.x

[20] Bradley KA , DeBenedetti AF , Volk RJ , Williams EC , Frank D , Kivlahan DR . AUDIT‐C as a brief screen for alcohol misuse in primary care. Alcohol Clin Exp Res. 2007;31(7):1208–17. 17451397 10.1111/j.1530-0277.2007.00403.x

[21] Heckathorn DD . Respondent‐driven sampling ii: deriving valid population estimates from chain‐referral samples of hidden populations. Soc Problems. 2002;49(1):11–34.

[22] Dunbar R . How many friends does one person need? Dunbar’s number and other evolutionary quirks. Cambridge, MA: Harvard University Press; 2010.

[23] US Centers for Disease Control and Prevention . *HIV treatment as prevention* [cited 2021 Feb 22]. Available from: https://www.cdc.gov/hiv/risk/art/index.html

[24] Gile KJ , Beaudry IS , Handcock MS , Ott MQ . Methods for inference from respondent‐driven sampling data. Annu Rev Stat Appl. 2018;5:65–93.

[25] Yauck M , Moodie EEM , Apelian H , Fourmigue A , Grace D , Hart T , et al. General regression methods for respondent‐driven sampling data. 2020 [cited 2021 Feb 23]. Available from: https://arxiv.org/abs/2012.00457 10.1177/09622802211032713 PMC8424528 34319832

[26] Hart T , Moore DM , Noor SW , Lachowsky N , Grace D , Cox J , et al. Prevalence of HIV and sexually transmitted and bloodborne infections (STBBI), and related preventive and risk behaviours, among gay, bisexual and other men who have sex with men in Montreal, Toronto and Vancouver. Results from the Engage Study. 2021 Can J Pub Health (in press). 2020. 10.17269/s41997-021-00546-z PMC8210738 34142353

[27] Armstrong HL , Wang L , Zhu J , Lachowsky NJ , Card KG , Wong J , et al. HIV testing among a representative community sample of gay, bisexual, and other men who have sex with men in Vancouver, Canada. AIDS Behav. 2019;23(2):347–58. 30145708 10.1007/s10461-018-2259-2 PMC7583640

[28] Public Health Agency of Canada . Estimates of HIV incidence, prevalence and Canada’s progress on meeting the 90‐90‐90 HIV targets. P.H.A.o. Canada., Editor. Ottawa, Canada: Government of Canada; 2018.

[29] Public Health Agency of Canada . *M‐track: enhanced surveillance of HIV, sexually transmitted and blood‐borne infections, and associated risk behaviours among men who have sex with men in Canada. phase 1 report* . Ottawa, Canada: Public Health Agency of Canada; 2011.

[30] British Columbia Centre for Disease Control . *HIV Annual report* . 2017. 10.14745/ccdr.v43i01a01 PMC5757678 29770040

[31] Ontario HIV Epidemiology and Surveillance Initiative . *New HIV diagnoses 2017* . 2017 [cited 2020 July 24]. Available from: http://www.ohesi.ca/new‐hiv‐diagnoses‐2017/

[32] Venne S , Blouin K . *Portrait des infections transmissibles sexuellement et par le sang (ITSS) au Québec: Année 2016 (et projections 2017)* . 2017.

[33] Azar MM , Springer SA , Meyer JP , Altice FL . A systematic review of the impact of alcohol use disorders on HIV treatment outcomes, adherence to antiretroviral therapy and health care utilization. Drug Alcohol Depend. 2010;112(3):178–93. 20705402 10.1016/j.drugalcdep.2010.06.014 PMC2997193

[34] Roth EA , Cui Z , Rich A , Lachowsky N , Sereda P , Card K , et al. Repeated measures analysis of alcohol patterns among gay and bisexual men in the momentum health study. Subst Use Misuse. 2018;53(5):816–27. 29172871 10.1080/10826084.2017.1388259 PMC6138047

[35] Moore DM , Armstrong HL , Bacani N , Skakoon‐Sparling S , Noor S , Lachowsky NJ , et al. Examining differential success in participant recruitment using respondent‐driven sampling (Rds) in a Canadian multi‐site study of gbMSM. In 28th Annual Canadian Conference on HIV/AIDS Research. Saskatoon, Canada; 2019.

[36] BC Centre for Excellence in HIV/AIDS . BC Minister of Health announces swift progress on PrEP program. 2018 [June 2018]. [cited 2020 Sep 22]. Available from: http://cfenet.ubc.ca/news/forecast/bc‐minister‐health‐announces‐swift‐progress‐prep‐program

[37] Cox J , Apelian H , Moodie EEM , Messier‐Peet M , Hart TA , Grace D et al. HIV pre‐exposure prophylaxis (PrEP) use among Urban Canadian gay, bisexual and other men who have sex with men for whom PrEP is clinically recommended: baseline results from the engage cohort study. CMAJ Open. 2021. (in press). 10.9778/cmajo.20200198 PMC8177951 34021010

